# Fungicides have complex effects on the wheat phyllosphere mycobiome

**DOI:** 10.1371/journal.pone.0213176

**Published:** 2019-03-20

**Authors:** Kamilla Knorr, Lise Nistrup Jørgensen, Mogens Nicolaisen

**Affiliations:** Department of Agroecology, Faculty of Science and Technology, Aarhus University, Slagelse, Denmark; Universita degli Studi di Pisa, ITALY

## Abstract

Effects of fungicide treatments on non-target fungi in the phyllosphere are not well known. We studied community composition and dynamics of target (*Puccinia striiformis*) and non-target fungi in wheat that was heavily infected with yellow rust. Mycobiotas in bulk leaf samples and individual leaves were studied by metabarcoding targeting the internal transcribed spacer-1 (ITS1) region of the ribosomal DNA. The amount of yellow rust in individual samples was quantified by qPCR (quantitative PCR). In addition, septoria tritici blotch (*Zymoseptoria tritici*), powdery mildew (*Blumeria graminis*), tan spot (*Pyrenophora tritici-repentis*), and yellow rust (*P*. *striiformis*) were visually evaluated. We showed how fungal communities were affected by three different broad-spectrum fungicides that had been applied at different timings and doses to control *Puccinia striiformis*. We showed that fungal content was relatively constant even after fungicide treatments. Principal component analysis demonstrated that communities from fungicide-treated plots could be separated from the communities in non-treated plots. We observed effects of fungicide treatments on fungal communities using different dose, timing and products. Some fungi, including the target organism *P*. *striiformis* were effectively controlled by most of the fungicide applications whereas some yeasts and also *P*. *tritici-repentis* increased after treatments. We demonstrated the feasibility of using metabarcoding as a supplement to visual assessments of fungicide effects on target as well as non-target fungi.

## Introduction

Fungicide treatments are common control strategies used to manage fungal pathogens in arable crop plants. Apart from reducing target pathogens, effects of fungicides on non-target fungi in the phyllosphere have been observed in several crops such as grapevine [1, 2], mango [3], and wheat [4, 5].

Yellow rust (*Puccinia striiformis*) is one of the most important diseases in wheat causing yield range from 1–5% but in cases with severe loss and reduced grain quality [6]. In most wheat growing regions crop losses due to yellow rust attacks reductions in the range of 25–50% are seen [6–8]. Fungicides are typically applied to reduce potential losses caused by yellow rust and other fungal pathogens in wheat fields. Based on data from variety trials Jørgensen et al. [7] estimated that average yield gains from use of fungicides range from 0.5 to 1.5 tonnes ha^-1^ in Denmark to 1.5 to 2.5 tonnes ha^-1^ in England. Choice of fungicide, timing and dose are known to influence the control level of yellow rust as well as other pathogens in wheat [9–11].

Karlsson et al. [4] studied the phyllosphere mycobiome of fungicide treated and non-treated wheat by 454 pyrosequencing of the internal transcribed spacer 2 (ITS2) region. They observed that fungicide treatments had significant effects on the mycobiome on wheat leaves resulting in lower richness and evenness of operational taxonomic units (OTUs). Even though fungicides are known to effectively reduce target diseases significantly, an unclear picture appeared as no significant effects on the abundance of the wheat pathogens *Mycosphaerella graminicola*, *Blumeria graminis*, *Puccinia striiformis*, *Phaeosphaeria nodorum*, *Monographella* spp., and *Pyrenophora tritici-repentis* were found [4]. This observation was supported by Sapkota et al. [5] who studied effects of fungicide treatments on fungal communities on cereal leaves from winter wheat and winter and spring barley. In their study *Sporobolomyces roseus* and *Dioszegia hungarica* showed significant positive responses to fungicide treatment whereas *Alternaria* sp., *Cladosporium* sp., *Epicoccum nigrum*, *Phaeospharia* sp. and *Ascochyta* sp showed significant negative responses to fungicide treatment, but none of the fungicide targets (e.g. *Zymoseptoria tritici*) were significantly affected.

In this study, we used metabarcoding of the fungal internal transcribed spacer 1 (ITS1) region to analyse the mycobiota of winter wheat inoculated with yellow rust and then treated with fungicides to control the infection. We used three timings and a two-spray strategy (application at two timings with reduced dose: split treatment), and two dose rates of three different broad spectrum fungicides to study effects of fungicide treatment in detail. The fungicides included azoles (FRAC group 3, prothioconazole and epoxiconazole), which are ergosterol inhibitors [12], strobilurins (FRAC group 11, pyraclostrobin) and succinate dehydrogenase inhibitors (SDHI) (FRAC group 7, boscalid, fluxapyroxad, and bixafen) that both interferes with respiration in the mithocondrial complex [12–14]. We addressed the following research questions: do fungicide treatments affect non-target pathogens? do variations in fungicide dose rates and application timings affect mycobiota composition? can metabarcoding be used as a tool to assess fungicide effects on fungal populations?

## Materials and methods

### Description of field experiments and wheat leaf sampling

Twenty one fungicide treatment combinations (fungicide choice, dose and timing) were tested for their control of yellow rust in a field trial carried out in the susceptible winter wheat cultivar Baltimor (Table 1). In addition to fungicide treatments the trial included non-treated controls with three replicates. The field trial was carried out at Research Center Flakkebjerg (Aarhus University, Denmark) from October 2010 to September 2011 on a clay loam soil. The experimental design was a randomised block design with 3 replicates and a plot size of 22.5 m^2^. The trial site was artificially inoculated using spreader plants (cultivar Brigadier) inoculated with *P*. *striiformis* f.sp. *tritici* isolate PstS0 [15] in April (17^th^ and 18^th^), (growth stage (GS) 24–30). The isolate used for inoculation is known to be aggressive on the cultivar Baltimor. The infected spreader plants were brushed across the canopy using one pot per plot. The inoculation gave rise to an even and severe attack of yellow rust starting at the lower leaves in the beginning of May.

**Table 1:** Fungicide treatments.

Products	g active ingredients/ha in full dose (100%)	Dose product	Timing
Viverda	125 g epoxiconazole + 150 g pyraclostrobin + 350 g boscalid	75% 25%	All applied once at GS 37, 39 or 51
Adexar	125 g epoxiconazole + 125 g fluxapyroxad	75% 25%	
Aviator	200 g prothioconazole + 100 g bixafen	75% 25%	
Viverda	31 g epoxiconazole + 37 g pyraclostrobin + 87 g boscalid	25%	Applied twice at GS 37 + 51
Adexar	31 g epoxiconazole + 31 g fluxapyroxad	25%	Applied twice at GS 37 + 51
Aviator	50 g prothioconazole + 25 g bixafen	25%	Applied twice at GS 37 + 51

Three different fungicides consisting of mixtures of active ingredients with different modes of actions were tested using Viverda (50 g epoxiconazole + 60 g pyraclostrobin + 140 g boscalid/l), Adexar (62.5 g epoxiconazole + 62.5 g fluxapyroxad/l) and Aviator (150 g prothioconazole + 75 g bixafen/l) (Table 1). Each product was tested at two dose rates (25% and 75% of standard label rate). The chosen rates are commonly used for testing products for control of yellow rust in EU [11]. Each product were tested at three timings using one week intervals between treatments. All plots, except the plots receiving split treatments, were treated once: the first application was carried out at flag leaf emergence (GS 37; May 19^th^), one week before yellow rust attack developed on the flag leaves. The second application was applied at full flag leaf emergence (GS 39; May 25^th^) at a time with 0.5–1.0% severity of yellow rust on flag leaves. The third application was applied at the beginning of ear emergence (GS 51; June 1^st^) with 1–2% severity on flag leaves. In addition, all three fungicides were tested in a split treatment with 25% standard rate being applied at GS 37 and GS 51 (Table 1). The use of a split treatment is common agricultural practice to control yellow rust. Fungicides were applied with a self-propelled sprayer using low pressure (2.9 bar), Hardi flat fan nozzles (green ISO 015), and 200 l/ha.

The trial was assessed several times during the season, but for this study data and leaves from GS 77 (late milk stage; July 18^th^; 48 days after last spraying) were used. Disease assessments of all plots were carried out as per cent coverage of flag leaves by the individual disease symptoms giving an average score per disease based on a visual scoring 4–5 places in each plot. Disease assessments were made for septoria tritici blotch (*Zymoseptoria tritici*), powdery mildew of wheat (*Blumeria graminis*), tan spot (*Pyrenophora tritici-repentis*), and yellow rust (*Puccinia striiformis*). After disease assessments 15 flag leaves were randomly picked in each of the three replicate plots. The leaves from each replicate were pooled prior to being stored at -20°C for downstream molecular analysis. Leaves from the three replicate plots were further selected for single leaf analysis using 15 leaves each from two extreme disease situations. The plots selected for the single leaf analysis were chosen in order to represent a plot with disease symptoms (untreated) and a plot with a low level of disease symptoms (split treatment with Viverda) based on the visual evaluations in the field.

### DNA extraction

Wheat leaf samples (pools of 15 leaves or single leaves) were homogenized in liquid N_2_ with six steel beads using a Geno/Grinder 2000 (OPS Diagnostics, Bridgewater, NJ, USA). DNA extractions were done using the ‘Mag^™^ maxi DNA extraction kit’ (LGC Genomics, Teddington, UK, cat no. 40430) and with the KingFisher DNA purification system (Thermo Electron Corporation, Waltham, MA, USA) according to manufacturer’s instructions.

### Estimation of fungal DNA using qPCR

The DNA of *P*. *striiformis* and the total fungal DNA in each sample was estimated by use of real-time PCR. In all cases, PCR reactions were performed in duplicate. Genomic DNA from leaf samples was diluted 1:10 before PCR on a 7900HT Sequence Detection System (Applied Biosystems, Waltham, MA, USA).

qPCR for estimation of *P*. *striiformis* DNA was carried out in a total reaction volume of 12.5 μl consisting of 6.25 μl 2 × TaqMan Universal PCR Master Mix (Applied Biosystems, cat. no. 4444556), 125 nM FAM TAMRA probe PsFAM2 (FAM—TAA GAC TTG GTT GCA TGA TTT GAA AGA ATC ATT—TAMRA), 375 nM of each primer ITS1rustF10d (TGA ACC TGC AGA AGG ATC ATT A) and ITS1rustR3c (TGA GAG CCT AGA GAT CCA TTG TTA) [16], and 2.5 μl template DNA. PCR was performed using the following cycling protocol: 2 min at 50 °C; 95 °C 10 min; 40 cycles of 95 °C for 15 s and 60 °C for 1 min.

qPCR for estimation of total fungal DNA was carried out in a total reaction volume of 12.5 μl consisting of 6.25 μl 2× SYBR Green PCR Master Mix (Applied Biosystems, cat. No. A25742), 375 nM of each primer ITS1F (CTT GGT CAT TTA GAG GAA GTAA) [17] and ITS2 (CTG CGT TCT TCA TCG AT) [18], 0.5 μg/μl bovine serum albumin (BSA) and 2.5 μl template DNA. Genomic DNA from leaf samples was diluted 1:10. PCR was performed using the following cycling protocol: 2 min at 50 °C; 95 °C 10 min; 40 cycles of 95 °C for 15 s, 55 °C for 1 min, and 60 °C for 1 min followed by dissociation curve analysis at 60 to 95 °C. Standard curves were generated using pure fungal DNA. Five-fold dilution series of *P*. *striiformis* isolate DK22/99 [19] and *F*. *graminearum* isolate 1955 [20] for estimation of *P*. *striiformis* DNA and for total fungal DNA, respectively, were used. The amounts of fungal DNA in samples were calculated from cycle threshold (Ct) values using standard curves.

### PCR amplification and metabarcoding

To generate amplicons from the ITS1 region for 454 pyrosequencing, ITS1F and ITS2 were used as template-specific primers for fusion primer design as described in earlier papers [5, 21]. The two primers were tag encoded using the forward primer design 5’- CGT ATC GCC TCC CTC GCG CCA TCA G–MID -ITS1F–3’ and the reverse primer design 5’- CTA TGC GCC TTG CCA GCC CGC TCA G—ITS2–3’. Thirty-six 10-nucleotide Multiplex Identifier (MID) primer tags for identification of sample-specific reads after pooling were selected randomly from the list of recommended MID primer tags from Eurofins MWG GmbH (Ebersberg, Germany). Primers were synthesized at HPLC purity by Eurofins MWG GmbH.

PCR reactions contained 1 × GoTaq Colorless Reaction Buffer, 1.5 mM MgCl_2_, 0.2 mM each dNTP, 1 μM of each primer, 0.625 U of GoTaq DNA Polymerase (Promega Corporation, Madison, USA, cat. no. M300) and 1 μl of DNA template in a final volume of 25 μl. All amplifications were conducted in an Applied Biosystems 2720 Thermal Cycler (Life Technologies Corporation, Carlsbad, USA) using an initial DNA denaturation step of 94 °C for 5 min, followed by 35 cycles at 94 °C for 1 min, 53 °C for 1 min, 72 °C for 1 min, and a final elongation at 72 °C for 7 min. PCR products were analysed by electrophoresis in a 1.5% agarose gel.

DNA amounts were estimated by gel electrophoresis and by analysis on a NanoDrop ND 1000 spectrophotometer (Thermo Fisher Scientific Inc., Waltham, MA, USA) according to manufacturer’s instructions. Tagged PCR amplicons were pooled in approximately the same amounts; amplicons from the 72 bulk samples were combined in two pools of 36 samples and the 30 single leaf samples were combined in one separate pool. The bulk samples were randomised with respect to sequencing pool and MID primer tags and the 30 single leaf samples were randomly assigned to MID primer tags. The pooled amplicons were precipitated and re-dissolved in 10 μl TE buffer. The pooled amplicons were electrophoresed in 1.5% agarose gels, and a visible smear of PCR products at approximately 280–360 bp were cut from the gel and purified using QIAquick Gel Extraction Kit (Qiagen GmbH, Hilden, Germany, cat. no. 28115) according to manufacturer’s instructions. The three pools were sequenced by Eurofins MWG GmbH on a GS Junior 454 Sequencer (Roche Diagnostics) and sequence data were delivered as MID tag-sorted sequences.

### OTU-based sequence analysis

Filtering, clustering, and BLAST searches of sequence reads were performed using the CLOTU application [22]. Initially, reads were quality filtered by discarding reads in which primers and tag could not be identified and reads which were shorter than 150 bp. Remaining reads were clustered using BLASTclust at 97% similarity and 90% coverage. Singletons were subsequently omitted from the dataset.

From the BLASTclust analysis, an OTU table was constructed containing the abundance of reads in each OTU from each sample. The abundance data was normalized with respect to total fungal DNA in each sample as quantified by qPCR. Unless stated otherwise the normalised data was used in all subsequent analyses.

BLAST searches were performed manually in GenBank using a set of randomly selected reads from each OTU. Rarefaction, species accumulation curves and the non-parametric α-diversity estimator Chao1 were calculated using PAST 3.06 [23].

Sequence files and metadata from this study were deposited in the NCBI sequence read archive under the number SRP167081 and the bioproject number PRJNA498985.

### Statistical analyses

Responses of the most abundant core OTUs (OTU1-14), total fungal DNA and *P*. *striiformis* DNA to fungicide treatment, dose and timing were compared using ANOVA factorial analysis using either least significant difference with a 95% confidence interval (LSD_95_) or Tukeys HSD using the ARM software (http://www.gdmdata.com/). Both tests performed similarly and data from LSD_95_ were presented. Transformation of data was included when needed for obtaining normal distribution. The disease assessment data were treated as interval data, and data were normalized and arcsinh transformed prior to calculations. Heat maps, PCA and boxplots were made using PAST 3.06 [23].

## Results

### Metabarcoding data

The ITS1 primers that we used for metabarcoding do not amplify *Puccinia* spp.[5], therefore, yellow rust infection was quantified by qPCR. To assess the effects of fungicide treatments we collected data on yellow rust infections quantified by qPCR, fungal metabarcoding data and by visual assessments of known diseases.

From the wheat plots, 72 bulked leaf samples and 30 single leaf samples were studied. The samples represented differences in timing and dose of three fungicides along with untreated controls. After quality filtering and exclusion of singletons there were 179,081 reads from the bulk samples and 91,182 reads from individual leaf samples, a total of 270,263 reads. The reads were clustered at 97% identity into 40 non-singleton OTUs. Each sample contained an average of 2650 ± 581 reads (min. 1353, max. 4331) (S1 Table). Rarefaction and species accumulation curves for both bulk and single leaf samples showed adequate sequencing and sampling depth as curves approached a plateau (S1 Fig). Estimated OTU richness in bulk samples was highest in samples from Adexar treated plots (Chao1_0.95_ = 38.0), followed by Viverda (Chao1_0.95_ = 37.3), untreated controls (Chao1_0.95_ = 36.5) and lowest in samples from Aviator treated plots (Chao1_0.95_ = 31.0). The estimated OTU richness in single leaf samples differed somewhat with Viverda (Chao1_0.95_ = 27.0) treated leaves having a lower estimated richness than untreated controls (Chao1_0.95_ = 32.7).

### Fungal content in wheat leaves

We arbitrarily considered 14 OTUs as ‘core’ members of the fungal communities as they were present in almost all samples and represented 96.5% of the fungal reads. These core OTUs were selected for further analysis (Table 2). On basis of the abundance data, those OTUs could be divided in four groups: very highly abundant (> 20%, OTUs 1–3), highly abundant (4–10%, OTUs 4–7), medium (1–4%, OTUs 8–11) and low abundance (< 1%, OTUs 12–14). All wheat pathogens of importance were detected in the data were within this range: *Cladosporium* sp. (black head mold), *Zymoseptoria tritici* (septoria tritici blotch), *Didymella exitialis* (ascochyta leaf spot), *Lewia infectoria* (black head mold), *Pyrenophora tritici-repentis* (tan spot), *Microdochium nivale* (fusarium head blight), *Phaeosphaeria nodorum* (stagonospora nodorum blotch) and *Blumeria graminis* (powdery mildew). Six of the core OTUs represented basidiomycete yeasts (*Sporobolomyces* sp., *Cryptococcus victoriae*, *C*. *tephrensis*, *C*. *stepposus*, *Dioszegia hungarica*, *Udeniomyces pannonicus*) where all except some *Sporobolomyces* sp., sometimes causing black head mold, are considered non-pathogenic on wheat. In addition to these, a number of OTUs were frequently found in the data, among those were weak pathogens such as *Epicoccum nigrum* (black head mold) as well as several basidiomycete yeasts (S1 Table). Since *Puccinia striiformis* for unknown reasons was not detected using the primers for metabarcoding, we quantified this fungus by qPCR. *P*. *striiformis* DNA was below detection level in 25 of 72 bulk samples and in 16 of 30 single leaf samples (S1 Table). A similar distribution of OTUs was observed in the single leaf samples except for *Pyrenophora tritici-repentis* which was present in 12 of 15 of the non-fungicide treated samples (S1 Table).

**Table 2:** Identity and number of reads of the 14 most abundant OTUs in the dataset. Disease caused by each pathogen is noted.

OTU	No.sequences	BLASTId	Phylum	Genus (Anamorph/ teleomorph)	Disease in Wheat
**1**	69873	*Sporobolomyces s*p.	Basidiomycota	Sporobolomyces	Non-pathogenic/Black head mold
**2**	57435	*Cladosporium* sp.	Ascomycota	Cladosporium/Davidiella	Non-pathogenic/Black head mold/Black point smudge
**3**	52791	*Mycosphaerella graminicola*	Ascomycota	Zymoseptoria/Mycosphaerella	Septoria tritici blotch
**4**	27001	*Didymella exitialis*	Ascomycota	Ascochyta/Didymella	Ascochyta leaf spot
**5**	12984	*Lewia infectoria*	Ascomycota	Alternaria/Lewia	Non-pathogenic/Black head mold
**6**	12141	*Cryptococcus victoriae*	Basidiomycota	Cryptococcus	Non-pathogenic
**7**	11343	*Cryptococcus stephrensis*	Basidiomycota	Cryptococcus	Non-pathogenic
**8**	6847	*Pyrenophora tritici-repentis*	Ascomycota	Pyrenophora/Drechsler	Tan spot
**9**	5239	*Dioszegia hungarica*	Basidiomycota	Dioszegia	Non-pathogenic
**10**	4639	*Udeniomyces pannonicus*	Basidiomycota	Udeniomyces	Non-pathogenic
**11**	3064	*Microdochium nivale*	Ascomycota	Microdochium/Monographella	Pink snow mold/Fusarium head blight
**12**	2816	*Phaeosphaeria nodorum*	Ascomycota	Stagonospora/Phaeosphaeria	Stagonospora nodorum blotch
**13**	1267	*Cryptococcus stepposus*	Basidiomycota	Cryptococcus	Non-pathogenic
**14**	659	*Blumeria graminis*	Ascomycota	Blumeria	Powdery mildew

### Visual disease assessment correlates with pathogen abundance

Pearson correlation coefficients were calculated to determine if there were positive correlations between the visual disease assessments of yellow rust, septoria tritici blotch, and tan spot, and the amounts of *P*. *striiformis* (qPCR), *Mycosphaerella graminicola* and *P*. *tritici-repentis* (metabarcoding) DNA (S2 Table). Results showed that there was a strong and highly significant correlation (r^2^ = 0.688, P = 0.001) between the visual disease assessments of yellow rust and the qPCR quantification of *P*. *striiformis* (S2 Fig). Regarding field data on septoria tritici blotch and the metabarcoding data on *Z*. *tritici* there was a modest but highly significant correlation (r^2^ = 0.288, P = 0.001). There was no significant correlation between tan spot symptoms and *Pyrenophora tritici-repentis* DNA, possibly due to low amounts of the pathogen and disease symptoms being difficult to differentiate from symptoms caused by other necrotrophs. Powdery mildew was also on the list of diseases to be evaluated in the field, but no visual symptoms were detected at this relatively late assessment date, which is in good agreement with the low abundance of *Blumeria graminis* DNA detected by metabarcoding. Yield data from the trial (S3 Table) showed significant yield responses from all treatments. Depending on efficacy, dose and timings yields were improved by 1.5–2.3 tonnes/ha. Best control of yellow rust and yield responses was obtained from the split control strategies.

### Phyllosphere mycobiota is affected by fungicide choice, timing and dose

**Fig 1:**
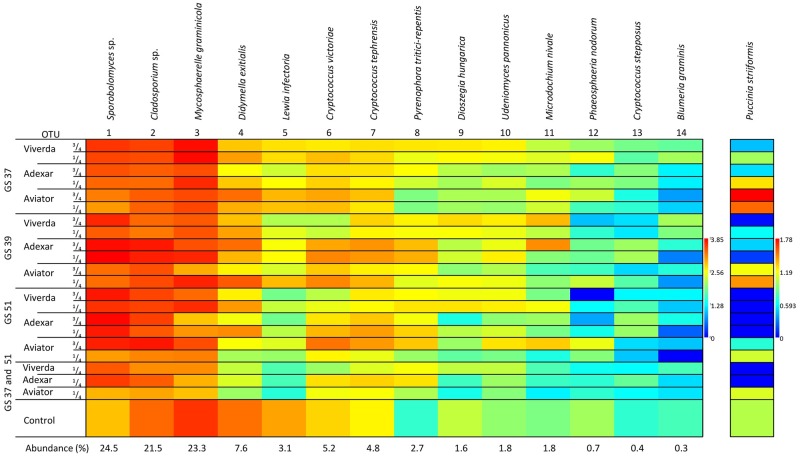
Heat matrix of the 14 most abundant OTUs in bulk samples, and *P*. *striiformis* DNA expressed as mean fungal DNA per treatment (log (x+1) transformed, n = 3). Red corresponds to high abundance and blue to low abundance.

**Fig 2:**
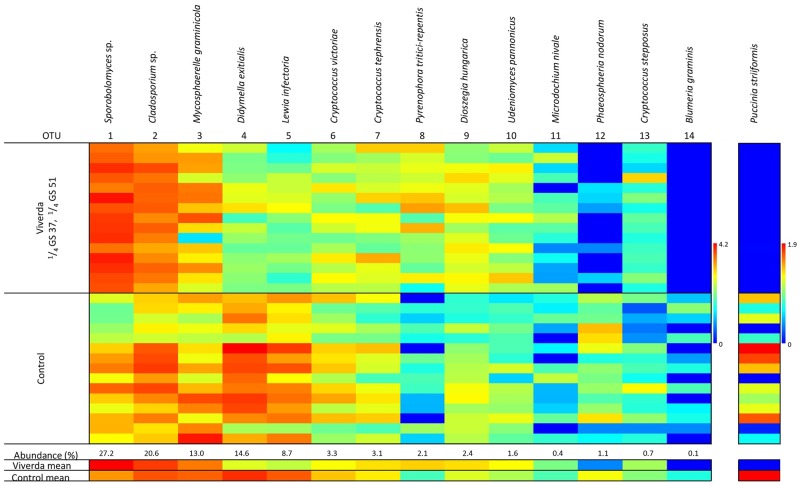
Heat matrix of the 14 most abundant OTUs in single leaf samples, and *P*. *striiformis* DNA expressed as mean fungal DNA per treatment (log (x+1) transformed, n = 3). Red corresponds to high abundance and blue to low abundance. Average of the two treatments in the last two rows.

**Fig 3:**
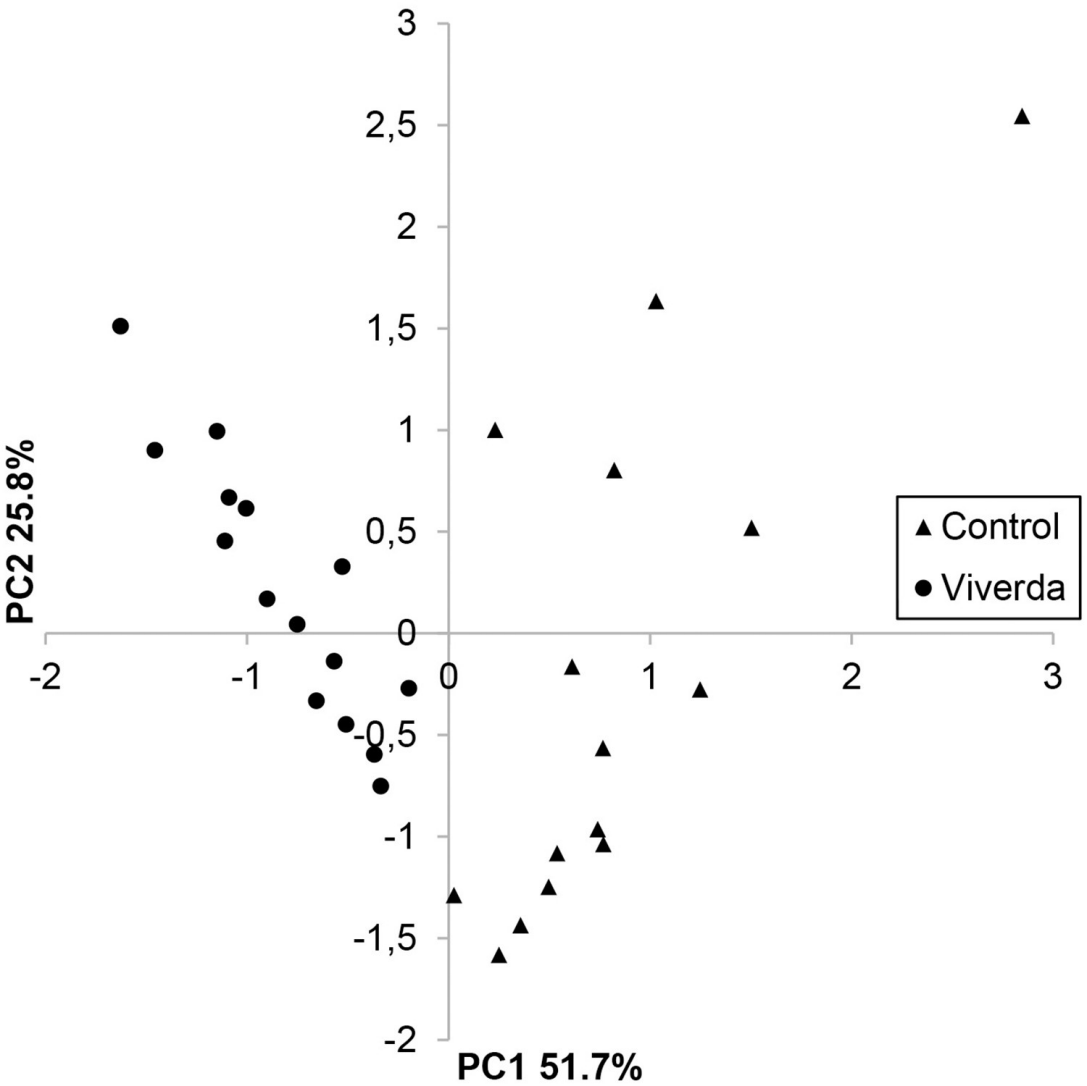
Distribution of fungal communities in single leaf samples according to treatment (untreated and split treatment with Viverda) using values normalized to total fungal biomass visualized by principal component analysis (PCA) on two axes, PC1 (51.7%) and PC2 (25.8%).

To visualise the fluctuations in the community composition with respect to fungicide treatments, a heat map of mean fungal DNA per treatment was made for bulk samples (Fig 1) and for single leaves (Fig 2). Effects of treatments on total fungal DNA, *P*. *striiformis* DNA, and DNA content of core fungi in the mycobiome are shown in Table 3 and S4 Table. qPCR quantification showed no significant differences in total fungal DNA between the three fungicides and non-treated controls, but early treatment and lower dose both resulted in a modest but significant increase in fungal DNA. Fungicide treatment generally affected fungal communities as could be observed in a PCA plot (Fig 3). Several effects of the different treatments on members of the fungal communities were observed (Figs 1 and 2 and Table 3). Similar to visual assessments (S2 Table), *P*. *striiformis* DNA responded to fungicide choice as Viverda and Adexar showed much better effect than Aviator for control of *P*. *striiformis*. Viverda was not efficient against *Blumeria graminis* as no difference from non-treated controls in bulked samples could be detected. (Fig 1 and Table 3). *Didymella exitalis* and *Lewia infectoria* were well-controlled by all three fungicides. In contrast *Pyrenophora tritici-repens* content increased dramatically after fungicide application. This increase in fungal DNA content after fungicide application was also observed for many yeasts, most notably *Sporobolomyces* sp., but also *Udeniomyces pannonicus* and *Cryptococcus* sp. The yeasts *Cryptococcus victoriae*, *Dioszegia hungarica* and *C*. *stepposus* all responded to choice of fungicide. Viverda was most effective against *C*. *victoriae* and the least effective against *Dioszegia hungarica*. Adexar was the least effective against *C*. *stepposus*. Timing of fungicide application had significant effects on *P*. *striiformis* DNA with lower DNA contents in the late treatments (GS 51). Also *Mycosporella graminicola*, *Didymella exitalis*, *Lewia infectoria* and *Phaeosphaeria nodorum* were most effectively controlled after late treatments. Finally, split treatments effectively controlled *D*. *exitalis* (Viverda and Aviator) and *L*. *infectoria* (all fungicides) (S4 Table). Dose had significant effects on relative amounts of a few OTUs but not in the case of *P*. *striiformis*. *Sporobolomyces* sp. increased with increasing dose whereas *M*. *graminicola* and *L*. *infectoria* decreased with higher fungicide doses.

**Table 3:** Summary of responses of total fungal DNA, relative abundance of OTU1-14, and *P*. *striiformis* DNA to fungicide choice, timing and dose. ANOVA factorial analysis followed by post hoc analysis (LSD, Student-Newman-Keuls) of means of variance using ARM software (http://www.gdmdata.com/) was used. Means followed by the same letter do not significantly differ (P = 0.05, LSD). META = metabarcoding.

		Total fungal Biomass		*Puccinia striiformis*		*Sporobolomyces* spp.		*Cladosporium* spp.		*Mycosphaerella graminicola*		*Didymella exitalis*		*Lewia infectoria*		*Cryptococcus victoriae*		Cryptococcus tephrensis		*Pyrenophora tritici-repens*		*Dioszegia hungarica*		*Udeniomyces pannonicus*		*Microdochium nivale*		*Phaeosphaeria nodorum*		*Cryptococcus stepposus*		*Blumeria graminis*	
Method		QPCR		QPCR		META		META		META		META		META		META		META		META		META		META		META		META		META		META	
FUNGICIDE MEANS																																	
1	Viverda 2.5 l/ha	6,07	a	0,81	b	3438,09	a	2671,58	a	3586,70	a	705,98	b	221,65	c	292,56	c	463,54	c	357,67	a	384,96	a	314,50	a	126,36	a	15,66	c	28,42	b	13,23	a
2	Adexar 2 l/ha	5,34	a	1,18	b	4294,89	a	3272,61	a	2410,93	b	639,36	b	200,51	c	777,34	a	818,79	a	483,93	a	108,21	b	196,08	b	102,93	a	22,32	bc	101,34	a	3,87	a
3	Aviator 1.25 l/ha	6,01	a	12,11	a	1744,14	b	2747,20	a	2388,82	b	572,57	b	341,60	b	949,94	a	620,50	b	205,86	b	112,69	b	159,18	b	68,77	a	57,99	ab	20,13	b	3,07	a
4	Untreated	6,44	a	7,17	a	671,67	c	1962,12	b	3562,20	a	1684,35	a	845,57	a	521,93	b	360,97	c	27,62	c	146,37	b	91,59	c	42,60	a	80,07	a	26,41	b	13,39	a
Standard deviation		1,520		0,336		0,199		10,247		14,543		0,303		0,250		5,425		5,006		5,756		0,242		0,224		0,596		0,603		2,101		0,801	
CV		25,480		51,143		6,008		19,934		26,737		10,416		9,875		21,987		21,282		37,994		10,955		9,996		31,337		38,486		33,343		88,799	
TIMING MEANS																																	
1	gs 37–39	7,21	a	7,48	a	1447,78	b	2249,04	a	3726,17	a	878,14	ab	494,84	a	588,05	a	446,93	b	143,95	b	166,36	ab	142,86	b	78,57	a	68,26	a	41,93	a	10,81	a
2	gs 45	5,34	b	3,49	b	2293,41	a	2832,45	a	3136,99	a	1028,92	a	389,39	a	658,77	a	655,51	a	306,84	a	209,92	a	220,44	a	85,79	a	40,20	a	38,83	a	10,93	a
3	gs 55	5,35	b	1,44	c	2555,67	a	2868,47	a	2126,24	b	593,25	b	198,04	b	579,57	a	566,31	ab	252,40	a	121,73	b	165,12	ab	72,19	a	16,64	b	36,88	a	2,61	b
Standard deviation		1,520		0,336		0,199		10,247		14,543		0,303		0,250		5,425		5,006		5,756		0,242		0,224		0,596		0,603		2,101		0,801	
CV		25,480		51,143		6,008		19,934		26,737		10,416		9,875		21,987		21,282		37,994		10,955		9,996		31,337		38,486		33,343		88,799	
DOSE MEANS																																	
1	75%	5,48	b	2,95	a	2342,83	a	2884,87	a	2536,92	b	721,56	a	293,25	b	578,49	a	581,29	a	250,93	a	146,26	a	172,57	a	101,27	a	28,62	a	36,36	a	7,39	a
2	25%	6,45	a	4,20	a	1775,80	b	2409,92	a	3411,39	a	914,55	a	386,69	a	638,84	a	525,20	a	208,16	a	179,46	a	173,96	a	61,05	a	45,03	a	42,12	a	6,59	a
Standard deviation		1,520		0,336		0,199		10,247		14,543		0,303		0,250		5,425		5,006		5,756		0,242		0,224		0,596		0,603		2,101		0,801	
CV		25,480		51,143		6,008		19,934		26,737		10,416		9,875		21,987		21,282		37,994		10,955		9,996		31,337		38,486		33,343		88,799	

### Single leaf samples show variable infection patterns

**Fig 4:**
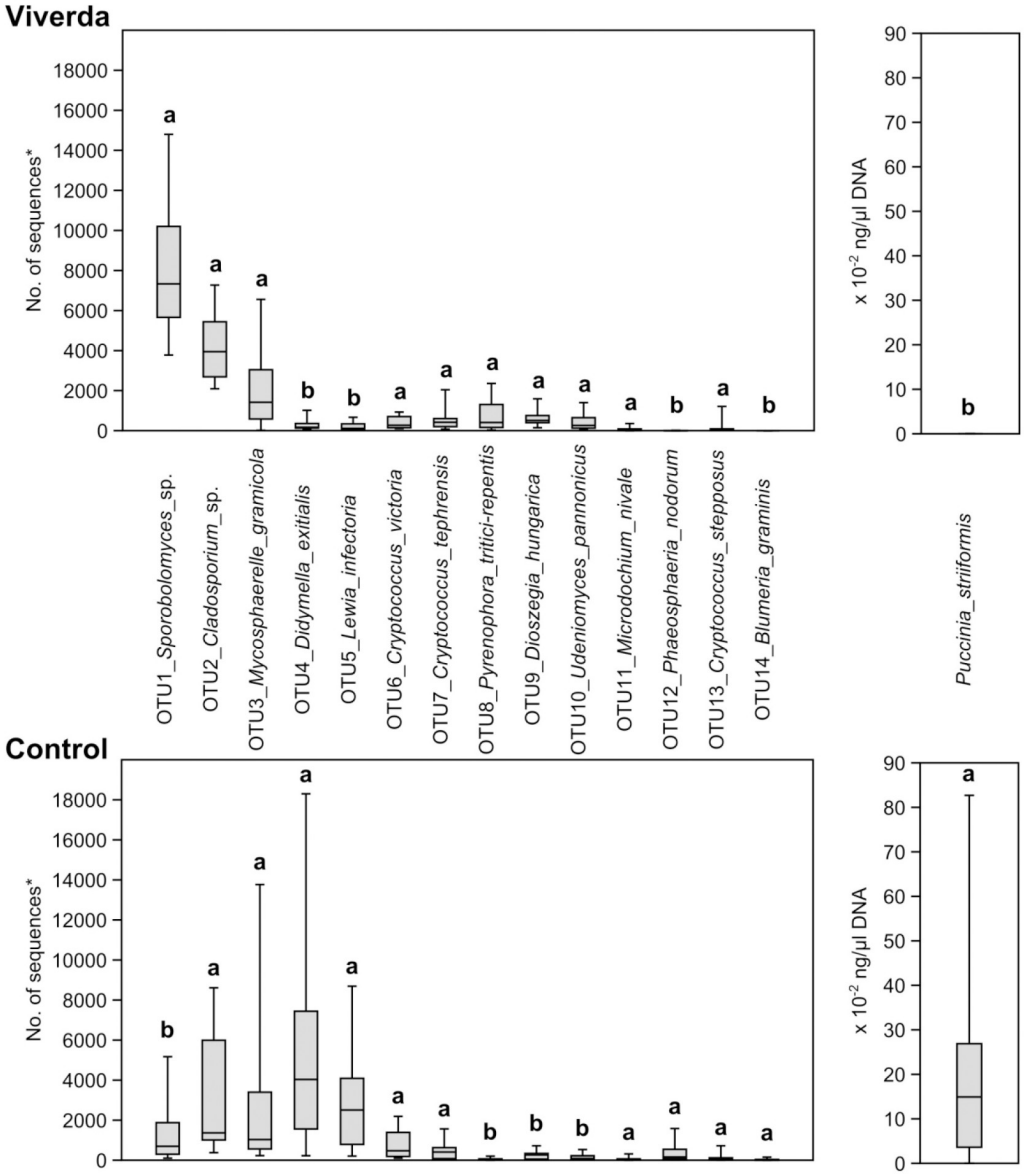
Distribution of the abundance of OTU 1–14 and *Puccinia striiformis* in the single leaf samples.

Using OTU data, a principal component analysis (PCA) showed a clear separation between the fungicide treated (Viverda split treatment) and the untreated controls with a higher variation between samples in the untreated group compared to the fungicide treated (Fig 3). A heat map of the community composition showed that there was a large variation in fungal communities between individual leaves from the same plots (Fig 2). Some fungi e.g. *Mycosphaerella graminicola* showed large variation of infection levels between leaves having received the same treatments, whereas others such as *Lewia infectoria* showed less variation between leaves. The distribution of OTUs 1–14 in the single leaves (Fig 4) confirmed the tendency of a higher variation within untreated samples compared to fungicide treated samples. *Sporobolomyces* sp., *Pyrenophora tritici-repentis*, *Didymella exitialis*, *Dioszegia hungarica*, and *Udeniomyces pannonicus* all had higher DNA contents, whereas *Lewia infectoria*, *Phaeosphaeria nodorum* and *Puccinia striiformis* all had lower DNA contents after treatment with Viverda (S4 Table).

## Discussion

This study addressed the effects of fungicides and their timing and dose on the phyllosphere mycobiota of wheat. The study used a systematic approach with a split plot design to test three fungicides in two dose rates and at three timings, and the resulting effects on fungal communities. We arbitrarily identified 14 core fungal species in our samples that were present in almost all samples. A similar set of core species was found in a recent study of the wheat grain mycobiome [24], except that the leaf pathogen *B*. *graminis* was absent in grain samples. A number of mycotoxin producing species of *Fusarium* were found in grains but not in leaves, reflecting the infection cycle of many members of this genus which is mainly through the head [25].

Interestingly, the total fungal DNA content was relatively constant in the different treatments indicating that spatial limits are restricting fungal colonisation of the phyllosphere (Table 3). A PCA plot comparing communities on treated vs. untreated single leaves showed marked effects of fungicides in our experiment (Fig 3). Yeasts like *Sporobolomyces* sp. or filamentous fungi with polycyclic infection cycles that are able to infect wheat late in the growing season after application of fungicides were able to exploit the available space. Among those polycyclic fungi were *Zymoseptoria tritici*, *Phaeosphaeria nodorum* and *Pyrenospora tritici-repentis* that all re-infect wheat after early fungicide applications. Likewise, *Fusarium* has been shown to increase after fungicide applications [26], probably due to its moderate sensitivity towards fungicides [27].

One of our initial interests was to test whether relative quantities obtained by metabarcoding of phyllosphere fungal communities in wheat correlated with the visually evaluated effects on known diseases at field level. When comparing the visual disease assessments of yellow rust and Septoria tritici blotch with the quantitative (qPCR) and semi-quantitative metabarcoding it was evident that if the disease level exceeds a certain threshold there is a positive correlation between molecular and visual methods. However, latent infections of e.g. *Zymoseptoria tritici* [28], the presence of dead fungal tissue, or the overgrowth of necrotic tissue by secondary fungi may have obscured the modest, albeit highly significant, correlation in the case of *Z*. *tritici*. In this specific study where severe attacks of *P*. *striiformis* dominated, it was difficult to visually separate other leaf diseases from necrotic attack of yellow rust late in the season. Sapkota et al. [5] observed a positive correlation between disease sympthoms of Septoria tritici blotch in wheat leaves and the relative number of *Z*. *tritici* reads obtained by metabarcoding. The pathogen *Phaeosphaeria nodorum* causing Stagonospora nodorum blotch in wheat was not visually evaluated at field level as the infection level was too low and symptoms were masked by Septoria tritici blotch. *P*. *nodorum* was detected by metabarcoding and the data revealed responses to treatment. Previously, it has been stated that quantification of DNA markers in samples does not suffice to describe biomass distribution; nucleus to biomass ratios may differ between taxonomic units and the number of DNA markers may differ in the genomes of different species [29]. However, comparison of identical OTUs between treatments is valid as these results show. Therefore, metabarcoding can add extra information to the control profile of different fungicides, where visual assessment can be difficult to apply with respect to separation of symptoms.

It is reasonable to assume that numbers of fungal species in crop plants would be reduced after fungicide treatments. However, species richness in plots and in single leaves was only moderately affected by fungicide choice. Only Aviator affected species richness negatively in the bulk samples and Viverda in the single leaf samples. Karlsson et al. [30] found higher fungal diversity in organically grown wheat. The non-significant effects in our experiments could be explained by the relatively small areas of untreated control plots that were surrounded by treated plots, compared to the much larger fields investigated in their experiment [30]. Other factors such as application of fertilizer might also have had an impact on the community diversity in organic fields compared to conventional fields in their experiments [30].

Fungal OTUs showed different trends in their reactions to fungicide treatments. Overall, three groups of OTUs based on their reactions could be established in the bulked as well as in the single leaf samples. One group generally reacted negatively to fungicide treatments such as *P*. *striiformis*, *Blumeria graminis*, *Phaeosphaeria nodorum* and *L*. *infectoria*; one group reacted positively to fungicide treatment (*P*. *tritici-repentis*, *Sporobolomyces* sp., *Cryptococcus* sp. and *Udeniomyces pannonicus*) and finally a group did not respond markedly to treatments. Pinto et al. [2] observed significant changes in microbial communities between fungicide treated and untreated grapevine. Treatments in this case consisted of several sprayings with a range of different fungicides. The three fungicides included in our study were all broad spectrum products, which, however, are known for slightly different efficacies for control of individual diseases. Viverda and Adexar were more efficient in controlling *Puccinia striiformis*, Aviator and Adexar were more efficient in controlling *Zymoseptoria tritici*, whereas Aviator was the most efficient fungicide against *P*. *tritici-repentis*. This corresponds to Viverda and Adexar both containing epoxiconazole which is known for its strength for control of rust diseases [11], while Aviator with its content of prothiconazole is known for its better control of *P*. *tritici-repentis*. Differences in the control profiles by fungicides have been shown in earlier studies. For example, Dickinson and Wallace [31] only found modest effects on phylloplane yeast populations by using tridemorph, whereas other fungicides such as benomyl and zineb were more effective against yeasts and filamentous fungi.

Timing of fungicide application affected the target organism *P*. *striiformis* resulting in less fungal DNA content in the late treatments and in split treatments. These later treatments provides a longer lasting effect than the earlier treatments [32]. Timing also affected *Lewia infectoria* and *Zymoseptoria tritici* DNA content with late applications as the most effective. Similar but weaker effects of timing could be observed in controlling *Didymella exitalis* and *Dioszegia hungarica*. This likely reflected that those two fungi are active late in the season. *L*. *infectoria*, *Microdochium nivale* and Z.*tritici* responded to application dose showing lower fungal DNA content with higher doses in most treatments. *Sporobolomyces* sp. was more abundant after spraying with high doses, probably caused by the fact that fungicide-insensitive fungi explored the available space that was left after fungicide application.

Although we did observe significant effects of fungicide application on pathogenic fungi, as discussed above, there was a striking resilience of fungal communities. This could be caused by fungi recovering from the time of treatment to the time of sampling, but could also be caused by technical issues such as amplification of DNA from dead fungal material. Similarly, Perazzolli et al. [1] found that penconazole treatment of grapevine did not significantly affect the epiphytic mycobiota in the phyllosphere compared to untreated controls at the same location. Also in strawberry, only few differences were found in the microbiota when comparing organic and conventional growing systems, in this case possibly due to no direct contact between foliar fungicides and the examined fruits [33].

The present study demonstrated that metabarcoding is a novel method that has potential to support the evaluation of efficacy profiles of fungicide treatments on a large number of target and non-target organisms including low abundance pathogens that cannot be evaluated at field level. In that way metabarcoding can facilitate a much more complete picture of the effects of fungicide treatments compared to visual assessments. Finally, although not the scope of this paper, studies like this may point to fungal species that act as antagonists against pathogens: the high diversity of non-pathogenic fungi and the limited space available on the leaf surface might fuel searches for biological control agents [34].

## Supporting information

S1 Table Read distribution in the samples, and total fungal and *P*. *striiformis* DNA. E = early treatment GS 37; I = intermediate treatment GS 39; L = late treatment GS 51; L, S = split treatment GS 37 and 51.
(XLSX)

S2 Table Read distribution and visual assessments. Read distribution normalized with total fungal DNA amount in each sample and visual assessments of disease severity (septoria tritici blotch, powdery mildew, tan spot and yellow rust measured as % leaf covered with symptoms.
(XLSX)

S3 Table Average yield and yield increases. Severity of symptoms of yellow rust and septoria tritici blotch in the different treatments. Average of three replicates.
(XLSX)

S4 Table Response of OTU1-14 and *P*. *striiformis* to fungicide choice, timing and dose. ANOVA factorial analysis followed by post hoc analysis (LSD, Student-Newman-Keuls) of means of variance using ARM software (http://www.gdmdata.com/).
(XLSX)

S1 Fig Rarefaction and species accumulation curves. Rarefaction curves for bulk (a) and single leaf (b) samples and species accumulation curves for bulk (c) and single leaf (d) samples; both based on fungicide treatment. Error bars indicate 95% confidence intervals.
(TIF)

S2 Fig Fungal DNA of *P*. *striiformis* and *Z*. *tritici* (ng/μl) plotted against visual assessments (per cent leaf coverage). (TIF)
