# Supplementary figures and images for: Fungicides have complex effects on the wheat phyllosphere mycobiome
Source: PLoS One. 2019 Mar 20;14(3):e0213176. doi: 10.1371/journal.pone.0213176 (PMC6426229; doi:10.1371/journal.pone.0213176)

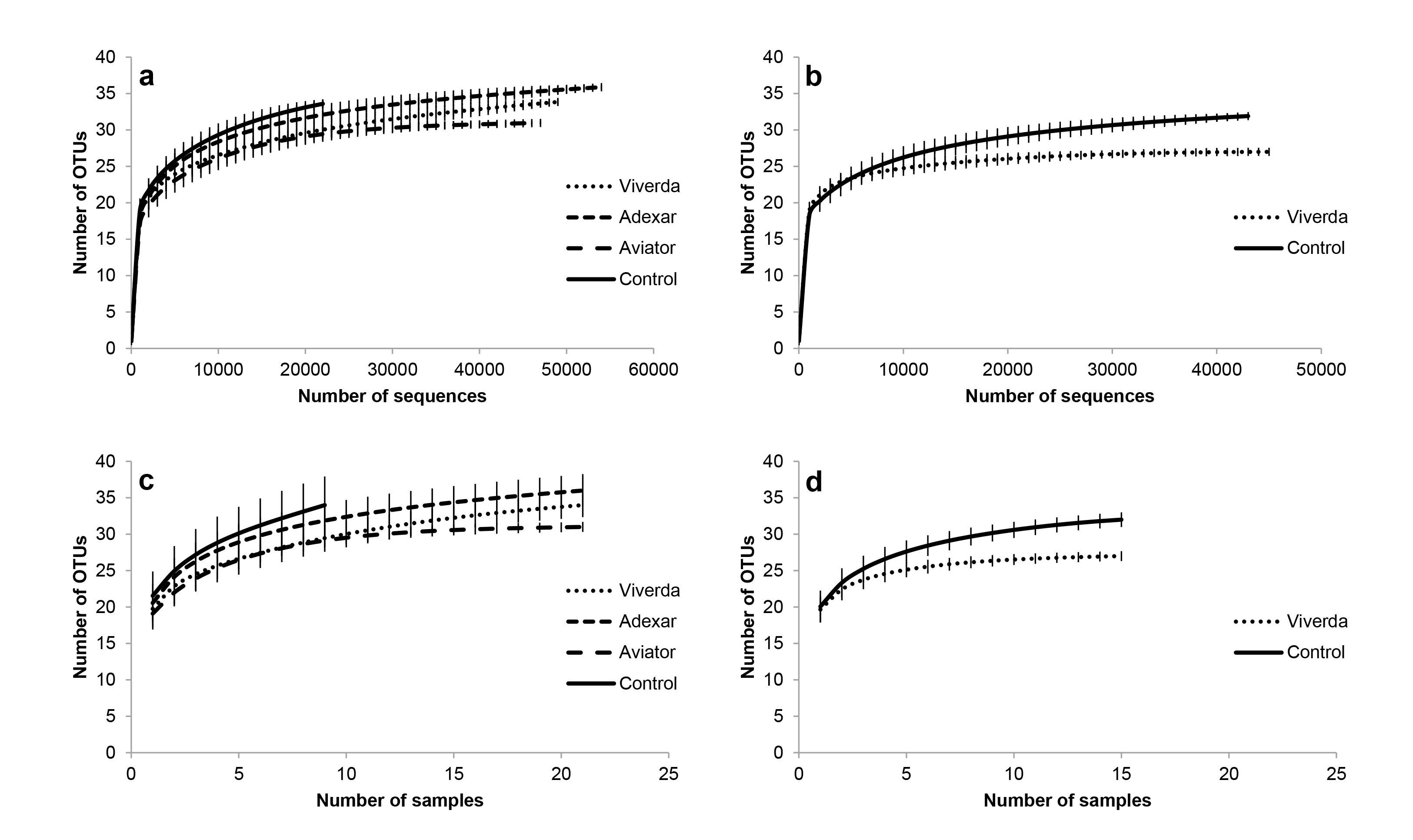

Supplement: S1 Fig — Rarefaction curves for bulk (a) and single leaf (b) samples and species accumulation curves for bulk (c) and single leaf (d) samples; both based on fungicide treatment. Error bars indicate 95% confidence intervals. (TIF) [file pone.0213176.s005.tif]

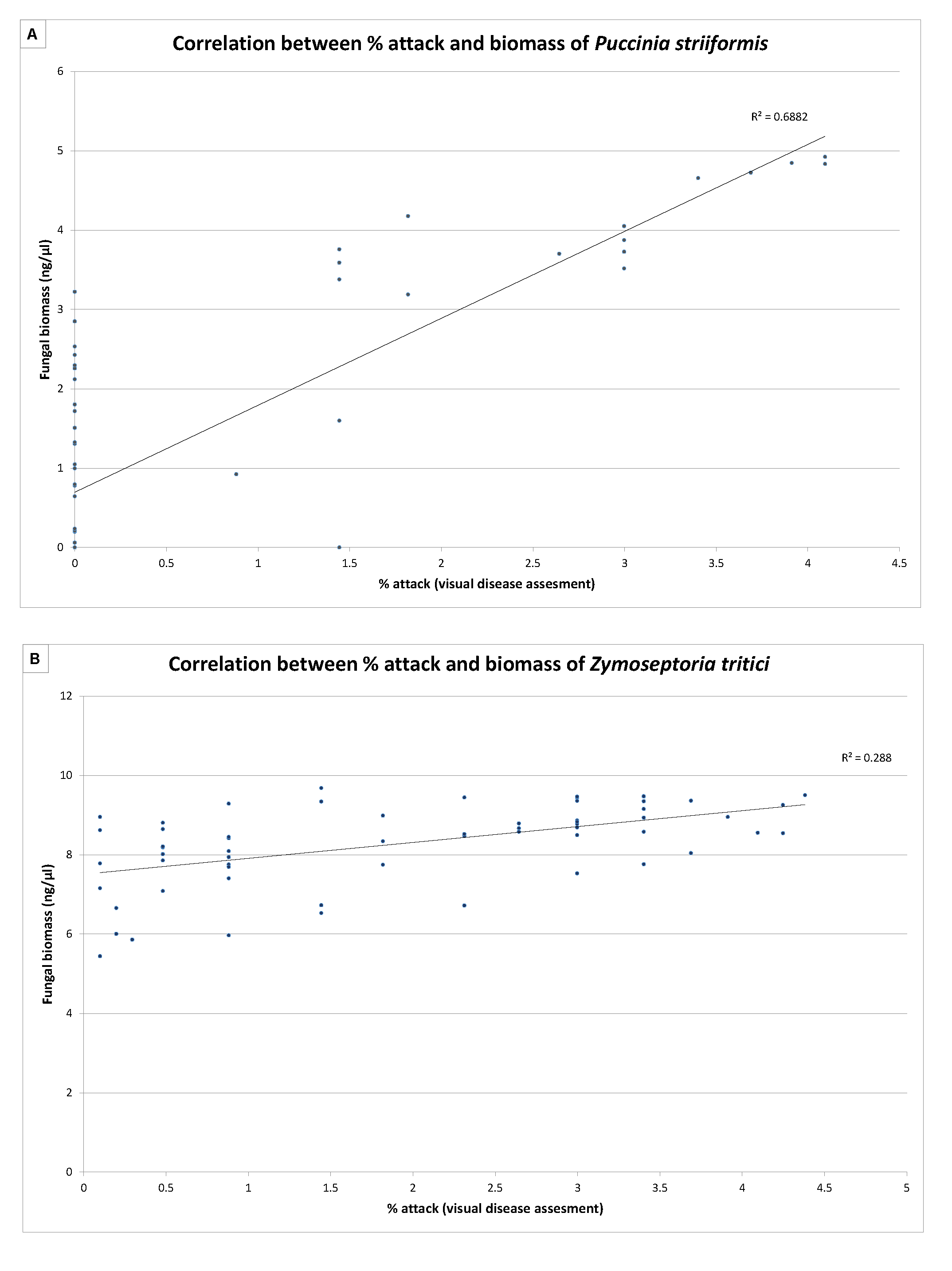

Supplement: S2 Fig — (TIF) [file pone.0213176.s006.tif]
